# Homeoprotein DLX4 expression is increased in inflammatory breast cancer cases from an urban African-American population

**DOI:** 10.18632/oncotarget.25790

**Published:** 2018-07-27

**Authors:** Jaehong Jeong, Tammey J. Naab, Aileen I. Fernandez, Martin S. Ongkeko, Kepher H. Makambi, Jan K. Blancato

**Affiliations:** ^1^ Department of Oncology, Georgetown University Medical Center, Washington DC 20057, USA; ^2^ Comprehensive and Integrative Medicine Institute, Daegu 42473, South Korea; ^3^ Department of Pathology, Howard University Hospital, Washington DC 20059, USA; ^4^ Department of Pathology, Georgetown University Medical Center, Washington DC 20057, USA; ^5^ Department of Biostatistics, Bioinformatics, and Biomathematics, Georgetown University, Washington DC 20057, USA

**Keywords:** DLX4, IBC, African-American, IHC, homeoprotein

## Abstract

Protein expression of Distal-less homeobox 4 (DLX4) was analyzed in inflammatory breast cancer (IBC) cases from an African-American (AA) population to determine if a) DLX4 gene over expression exists in this cohort and b) if the overexpression is associated with breast cancer clinicopathological characteristics (ER, PR, HER2, triple-negative). Twenty-nine blocks of formalin-fixed paraffin-embedded (FFPE) tissue from well-characterized human IBC cases were used for immunohistochemical staining (IHC). IHC results were assigned an intensity and percentage score. Percentage scores were assigned as 0, 1, 2, 3, or 4 and intensity scores were assigned 0, 1+, 2+ or 3+. For the analysis of the IHC, a percentage score of 3 or 4 and an intensity score of 2+ or 3+ were categorized as high. Chi-square or Fisher's exact tests were used to compare the high and low groups.

In this cohort, 89.7% (26 out of 29) of IBC cases showed high percentages of positive cells staining for the DLX4 protein, while 40.0% (12 out of 30) of normal breast tissue from reduction mammoplasty cases demonstrated DLX4 expression (*p* < 0.01). In IBC patients, 65.5% of cases showed a high level of staining intensity, compared to 20.0% of normal breast tissues (test, *p* = 0.001). Intensity to DLX4 was higher in the HER2 negative status (78.3%) than the HER2 positive status (16.7%) (test, *p* = 0.011).

DLX4 expression is higher in the IBC cases in this study of an urban AA population than in normal breast tissue cases. HER2 negative status is positively associated with high intensity of DLX4.

## INTRODUCTION

Inflammatory breast cancer (IBC) is one of the most aggressive subtypes of breast cancer [1]. As per the 8th edition of the American Joint Commission on Cancer (AJCC) Staging Manual a tumor is considered pT4 if it is of any size and is a direct extension to the chest wall and/or to the skin (ulceration or skin nodules) [2]. The AJCC and the College of American Pathologists (CAP) Cancer Protocol for invasive breast carcinoma, define inflammatory carcinoma as being pT4d. It is associated with histopathological findings but also requires a specific clinical presentation: an indurated peau d’ orange appearance, in which one-third or more of the breast has diffuse erythema and edema. Often, carcinomas that present within lymphatic spaces in the dermis correlate with the clinical features of inflammatory carcinoma are also classified as pT4d [2, 3]. IBC is rare and at presentation is both aggressive and progresses rapidly, with 30% of patients having distant metastases at the time of diagnosis [4]. The median overall survival of women with IBC is less than 4 years [5].

According to the Surveillance, Epidemiology, and End Results (SEER) 9 Registries from 1988–2000, though IBC accounted for only 2% of all breast cancer incidences, 7.0% of all breast cancer-specific deaths were due to IBC [6]. The American Cancer Society [7], reports that IBC is more common in African American (AA) women with an incidence of 10%, compared to 6% in Caucasian women, and 5% in other ethnic groups. A trend analysis of SEER data indicates that the incidence of IBC in AA women is significantly higher than in White women, and that AA women have lower survival than Caucasian women with IBC or non-IBC breast cancer [8]. Biological explanations for racial disparity in survival of all breast cancer subtypes are needed to improve this prognosis disparity.

For improved detection and treatment of this disease, it is not only necessary to have a better understanding of IBC characteristics, but also to identify new biomarkers. Previous studies have reported HER2- (Human epidermal growth factor receptor type 2) positive status in nearly half of all newly diagnosed IBC [9, 10], but few studies have identified HER2 receptor status as a prognostic factor. In 2008, Dawood, *et al.* [11] reported that HER2-positivity does not affect recurrence-free survival in IBC but that hazard of death is lower in HER2-positive IBC compared to HER2-negative IBC. Another study using the California Cancer Registry also reported HER2-positive IBC shows no statistically different breast-cancer specific-survival compared to HER2-negative IBC on unadjusted analysis, and that HER2-positive IBC has a slightly higher survival on adjusted analysis [12].

It is believed that the major biological factor that impacts the aggressive nature of IBC is the propensity for angiogenesis of the metastatic cells [13]. Large tumor emboli are observed in dilated dermal lymph vessels, which is a pathologic hallmark of IBC [14]. In addition to dissemination of tumor cells via the bloodstream, studies demonstrated intense angiogenesis and lymphangiogenesis in IBC with increased angiogenic factors including fibroblast growth factor and vascular endothelial growth factor receptor-2 (VEGFR-2) [15, 16]. The identification of these hallmarks may be advantageous in biomarker development for screening and treatment decisions.

Aberrantly expressed homeobox genes in malignant cells are important regulators in the metastatic process [17]. Altered expression of HOX genes is associated with development of acute lymphocytic leukemia and acute myeloid leukemia [18]. Studies have shown the role of homeobox genes in solid tumors as well; CDX2, HOXA5, ALX3 and HOXA7 genes are aberrantly expressed in colorectal cancer [19], breast cancer [20], neuroblastoma [21] and epithelial ovarian cancer [22], respectively.

This study focuses on Distal-less homeobox 4 gene (DLX4), which was first isolated among other members of the Distal-less family from the human placenta, and is mapped to human chromosome 17q21.33 [23]. DLX4, also called BP1, is a transcription factor normally expressed during early development and differentiation [23]. Mutations in the gene have been associated with structural birth defects. A single sequence variant in the DLX4 gene has been implicated as causal in bilateral cleft lip/cleft palate in a single family [24].

The DLX4 gene has two distinct splice variants, BP1 and DLX7, mapped to the same region on chromosome 17q21-22, that vary in their 5′ regions. BP1 is known to act as a putative repressor of human b-globulin, but splice variant DXL7 does not have any known repressor activity [25, 26]. They are both expressed in the myeloid lineage but distinct roles in leukemia have not been detailed [25]. It has been demonstrated that DLX4 is significantly expressed in prostate adenocarcinoma, with higher levels correlating with Ki-67 positive cells [27]. Earlier reports showed that over expression and/or gene amplification of the DLX4 gene may be associated with tumorigenesis in estrogen negative breast cancer [28, 29]. Interestingly, Man *et al.* [30] demonstrated that protein expression of DLX4 (referred to as BP-1), measured by immunohistochemistry (IHC), is significantly higher, both in intensity and percentage of cells, in IBC cases from the Inflammatory Breast Cancer Registry (IBCR) than in non-IBC cases. That study, showed that BP-1 protein levels were barely detectable in normal human breast tissues, while 21% of hyperplastic tissues, and 81% of invasive breast tissues showed high protein expression. The authors suggest that BP-1 may represent a signature marker for IBC and tumor aggression. However, here is no racial delineation provided for the subjects related to these compelling results [30]. In the present study, we aim to characterize DLX4 expression in IBC tissues from AA women. We used IHC to analyze DLX4 expression in both IBC tissues and in normal breast tissues from reduction mammoplasty, from AA women, while also looking at other clinic-pathological factors including Her2/Neu, estrogen receptor (ER) and progesterone receptor (PR) status.

## RESULTS

### Clinical characteristics of IBC cohort

There were a total of 59 samples in the study: 30 normal breast tissue, or controls from Caucasian women, and 29 IBC cases, from AA patients. Characteristics of the IBC patients and tumors are presented in Table 1. The median age was 59 years with a range of 32–82. Twenty-eight of the 29 patients were female and 1 was male. 82.8% of the IBC cases were stage pT4 tumors while 17.2% of the tumors were stage pT1, pT2, or pT3. 75.9% of the samples were graded 3 and 24.1% were grades 1 or 2. For receptor and HER2 status: 69% (20/29) were ER-positive and 31% (9/29) were ER-negative; 41.4% (12/29) were PR-positive, and 58.6%(17/29) were PR-negative; 20.7% (6/29) were HER2-positive and 79.3% (23/29) were HER2-negative; finally, 13.8% (4/29) were triple negative (IBC cases that were ER, PR and HER2- negative were considered triple negative breast cancer (TNBC), and non-triple negative breast cancer if any marker was positive) (Table 1). The 30 normal breast tissues from reduction mammoplasties and 29 IBC tissues were analyzed using IHC with a DLX4 variant 1 antibody. IHC results were assigned percentage and intensity score based on number of cells stained and intensity of positive staining, respectively.

**Table 1:** Characteristics of IBC patients analyzed for DLX4 expression in tissues

Demographic and clinical characteristics	
**Age, median (range)**	59 (32–82)
**Sex, *n* (%)**	
**Female**	28 (96.6)
**Male**	1 (3.4)
**Race, *n* (%)**	
**African-American**	29 (100.0)
**T Stage, *n* (%)**	
**pT1**	2 (6.9)
**pT2**	2 (6.9)
**pT3**	1 (3.4)
**pT4**	24 (82.8)
**N Stage, *n* (%)**	
**pNX**	5 (17.2)
**pN0**	3 (10.3)
**pN1**	6 (20.7)
**pN2**	6 (20.7)
**pN3**	9 (31.0)
Histologic grade*, *n* (%)	
**Grade 1**	1 (3.4)
**Grade 2**	6 (20.7)
**Grade 3**	22 (75.9)
**Estrogen receptor, *n* (%)**	
**Positive**	20 (69.0)
**Negative**	9 (31.0)
**Progesterone receptor, *n* (%)**	
**Positive**	12 (41.4)
**Negative**	17 (58.6)
**HER-2, *n* (%)**	
**Positive**	6 (20.7)
**Negative**	23 (79.3)
**Triple negative status, *n* (%)**	
**Positive**	4 (13.8)
**Negative**	25 (86.2)

### Immunohistochemical findings

**Figure 1:**
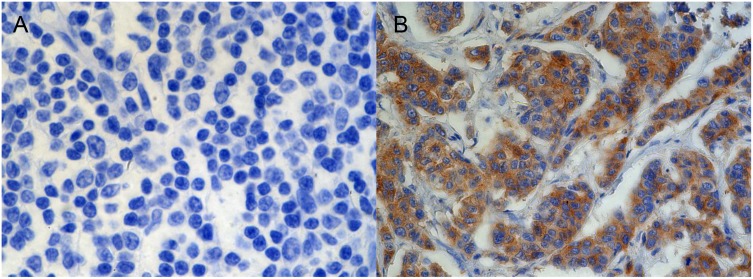
Representative images of negative (**A**) and positive (**B**) controls for immunohistochemical staining with DLX4, isoform 1 antibody (20×). (A) Tonsil tissue (B) Invasive moderately differentiated ductal carcinoma.

Negative and positive controls for the DLX4 antibodies, including a trial with PBS substituted for antibody on a FFPE-embedded human tonsil tissue are shown (Figure 1A). As a positive control, invasive moderately differentiated ductal carcinoma was stained with the DLX4 antibody. As expected, there was strong positive staining with a Nottingham score of 0.3 (Figure 1B). No DLX4 staining is seen in normal human fat tissue control (not shown).

**Figure 2:**
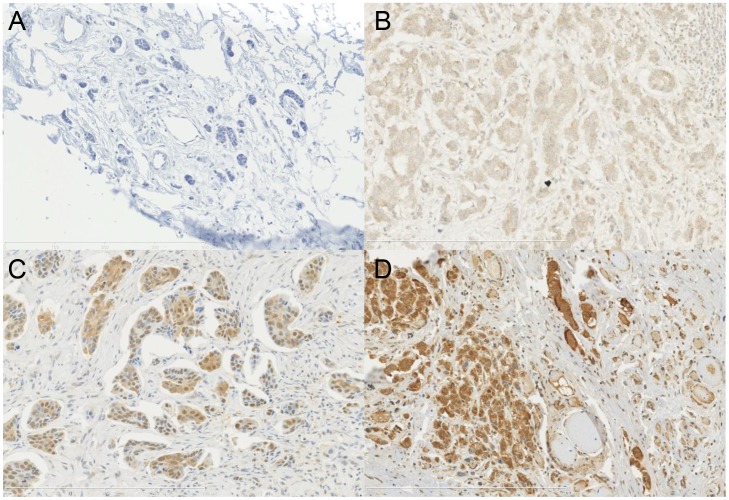
Immunohistochemical staining with DLX4, isoform 1 antibody in FFPE IBC tissues. Counterstain is H&E (20×) Representative images of intensity scores 0 (**A**), 1+ (**B**), 2+ (**C**), 3+ (**D**).

**Figure 3:**
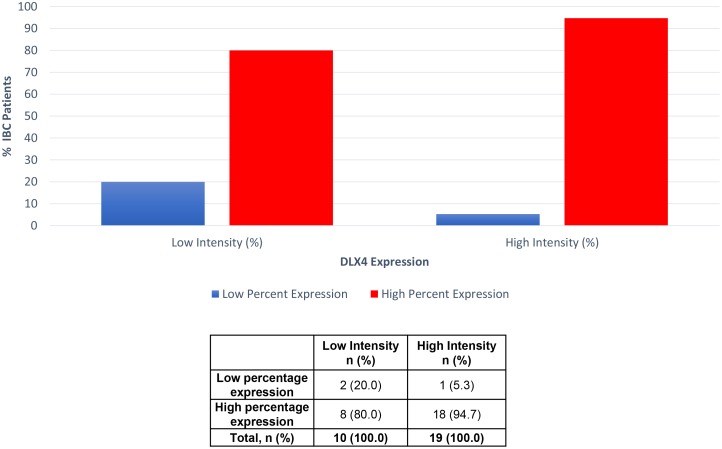
Percent cells expressing DLX4 by staining intensity level (%). 80% of the samples with low intensity scores, versus 94.7% of the samples with high intensity scores, showed high percent DLX4 expression

Percentage scores were assigned as 0, 1 (0%–25%), 2 (26%–50%), 3 (51–75%), 4 (76%–100%). Intensity scores were assigned 0, 1+, 2+ and 3+. Representative images of DLX4 IHC staining are provided (Figure 2). This includes negative staining (Figure 2A), as well as 1+, 2+ and 3+ intensity on IBC tissues (Figure 2B–2D, respectively), normal reduction mammoplasty not shown. Percent expression levels did not correlate with intensity of staining (Figure 3).

DLX4 comparative expression data for IBC and control tissues is provided (Table 2). In the categorized cohort, 89.7% (26 out of 29) of IBC cases showed a high percentage of positive cells staining for the DLX4 protein, while a lower percentage, 40.0% (12 out of 30), of normal breast tissue cases demonstrated DLX4 expression (chi-square test, *p* < 0.01). When assessing staining intensity, 65.5% (19 out of 29) of IBC cases showed a high level of intensity, while only 20.0% (6 out of 30) of normal breast tissues (chi-square test, *p* < 0.01) showed high staining intensity.

**Table 2:** Comparison of DLX4 protein expression (IHC) between IBC and controls

Percent positivity			
	Low *n* (%)	High *n* (%)	*P*-value
**Controls (*n* = 30)**	18 (60.0)	12 (40.0)	<0.001
**IBCs (*n* = 29)**	3 (10.3)	26 (89.7)	
**Staining intensity**			
	**Low** ***n* (%)**	**High** ***n* (%)**	***P*-value**
**Controls (*n* = 30)**	24 (80.0)	6 (20.0)	0.001
**IBCs (*n* = 29)**	10 (34.5)	19 (65.5)	

Comparison of DLX4 protein expression (IHC) between IBC and controls. Consecutive serial sections of normal breast tissues and IBC were scored for DLX4 protein expression, by IHC. Data were analyzed for correlations between. A significant association was observed between IBC and high DLX4 protein expression (IHC) in both of percent positivity and staining intensity. All of the *p*-value results were obtained from Chi-square test.

### Older patients showed higher intensity staining of DLX4

We looked at associations of clinicopathology characteristics and DLX4 percent positivity (Table 3) and staining intensity (Table 4). Most the IBC tissues (89%) showed positive staining for DLX4 (Table 3, percent). There was no significant difference in age between low and high percent positivity DLX4 expression (median age: 57.0 in low expression, 59.8 in high expression, *p* = 0.389). There were no statistical associations between non-TNBC versus TNBC status and DLX4 expression (Table 3). Interestingly, high staining intensity, indicating increased DLX4 expression, was associated with an older patient age in IBC (median age: 62.9 in high staining intensity, 52.0 in low staining intensity, *p* = 0.019) (Table 4). There were no significant associations between DLX4 intensity and hormonal status. However, there was a statistically significant association between HER2 status and DLX4 intensity staining. 78.3% (18/23) of the HER2-negative IBC cases showed high DLX4 intensity, compared to 16.7% (1/6) in HER2 positive IBC cases (*p* < 0.05, Fisher's exact test) (Table 4).

**Table 3:** Associations between clinicopathologic characteristics and DLX4 protein expression (percent positivity)

Clinicopathologic Characteristics			
	Low *n* (%)	High *n* (%)	*P*-value
**Number of cases**	3 (10.3)	26 (89.7)	
**Age (median)**	57.0	59.8	0.389_a_
**Estrogen receptor**			
**Positive**	2 (10.0)	18 (90.0)	>0.999
**Negative**	1 (11.1)	8 (88.9)	
**Progesterone receptor**			
**Positive**	2 (16.7)	10 (83.3)	0.553
**Negative**	1 (5.9)	16 (94.1)	
**HER2**			
**Positive**	1 (16.7)	5 (83.3)	0.515
**Negative**	2 (8.7)	21 (91.3)	
**Triple negative status**			
**Positive**	1 (25.0)	3 (75.0)	0.371
**Negative**	2 (8.0)	23 (92.0)	

^*^In all 29 cases of IBC tissues, associations between hormone-receptors or HER2 status and DLX4 expression (IHC) have been analyzed. It includes ER, PR, HER2 and TNBC. IHC scores were defined in the materials and methods section. For the analysis of the protein expression (IHC), a percentage score of 3 or 4 was considered high, and the score of 0, 1 or 2 low. All of the *p*-value results except _a_ were obtained from Fisher's exact test. The *p*-value result of a were from Mann–Whitney *U* test. *p < 0.05, IBC = inflammatory breast cancer; HER2 = human epidermal growth factor receptor type 2.

**Table 4:** Associations between clinicopathologic characteristics and DLX4 protein expression (staining intensity)

Clinicopathologic Characteristics			
	Low *n* (%)	High *n* (%)	*P*-value
**Number of cases**	10 (34.5)	19 (65.5)	
**Age (median)**	52.0	62.9	0.019_a_^*^
**Estrogen receptor**			
**Positive**	5 (25.0)	15 (75.0)	0.205
**Negative**	5 (55.6)	4 (44.4)	
**Progesterone receptor**			
**Positive**	4 (33.3)	8 (66.7)	0.615
**Negative**	6 (35.3)	11 (64.7)	
**HER2**			
**Positive**	5 (83.3)	1 (16.7)	0.011^*^
**Negative**	5 (21.7)	18 (78.3)	
**Triple negative status**			
**Positive**	1 (25.0)	3 (75.0)	>0.999
**Negative**	9 (36.0)	16 (64.0)	

^*^In all 29 cases of IBC tissues, associations between hormone-receptors or HER2 status and DLX4 expression (IHC) have been analyzed. It includes ER, PR, HER2 and TNBC. IHC scores were defined in the materials and methods section. For the analysis of the protein expression (IHC), an intensity score of 2+ or 3+ was categorized as high, and the score of 0 or 1+ low. All of the *p*-value results except _a_ were obtained from Fisher's exact test. The *p*-value result of a were from Mann–Whitney *U* test. *p < 0.05, IBC = inflammatory breast cancer; HER2 = human epidermal growth factor receptor type 2.

## DISCUSSION

Although the mechanism and pathway of DLX4 and its involvement in IBC maintenance and progression remains unknown, it has been suggested to be a marker for tumor aggressiveness [12]. In this study, we observed that the percentage and intensity of DLX4 immuno-staining is significantly greater in IBC tissues than in normal reduction breast mammoplasty tissues. This is a novel study looking at DLX4 in IBC breast tissues from an urban AA population that helps set the stage for further discussion and studies. Our findings are consistent with the previous reports that DLX4 is over-expressed in IBC [30]. Interestingly, our study also shows a significant association between HER2-negative status and high DLX4 positive protein expression- an association not previously reported. It is important to note that our cohort has very few HER2+ samples with only 20.7% (6 out of 29 IBC cases) being HER2-positive. Previous studies have reported a higher incidence of HER2 overexpression in IBC cases (52%) [21, 22], though neither study reported HER2-positive status as a significant prognostic factor for survival in IBC patients. Furthermore, a study done using the California Cancer Registry Data also showed that higher IBC incidence rate is not dependent on HER2-status, as IBC patients had lower survival rates compared with other patients, regardless of HER2 positivity [29].

A study of non-IBC Caucasian breast cancer lesions and metastases by Cavalli *et al.* [28], used *Fluorescent In Situ Hybridization* (FISH) to show that at the DNA level HER2 and DXL4 gene expression are correlated. Torresan, *et al.* [31] used a similar gene copy number approach to look at the 17q region in invasive carcinomas from a largely Caucasian population. The authors hypothesized that there was an association between amplification of HER2 and DLX4 in breast cancer since both HER2 and DLX4 genes are closely mapped on the long arm of human chromosome 17 but found no obvious gene co-amplification between the two. These two studies suggest that increased DLX4 gene copy number is not associated with amplification of HER2, making the mechanism of gene amplification unknown. This suggests that other factors, such as DLX4 and microenvironment function are contributors.

The association between highly expressing DLX4 homeoprotein and ER-negative breast cancer remains controversial, although it was not a factor in our study. Fu, *et al.* [32] previously reported that DLX4 over expression may be associated with ER-negative breast cancer and shows induction of an aggressive phenotype when DLX4 is overexpressed *in vitro*. However, a more recent study on breast cancer cases in a South African population did not find any association between DLX4 with ER protein expression [33]. Our study on AA patient samples, did not show a significant difference in DLX4 expression levels in ER, or PR, negative tumors. Furthermore, there was no statistical difference in DLX4 expression levels in TNBC in our IBC samples from our urban AA population. We did show for the first time, that in this cohort higher expression of DXL4 homeoprotein is related to a later age of diagnosis. This study shows that increased expression of the DXL4 homeoprotein is independent of hormone receptor status and associated with HER2-negative IBC tumors of AA patients.

DLX4 has been shown to be a potentially useful marker for the aggressive breast cancer subtype, IBC. Investigations on this gene and protein product, however, have employed assays for different endpoints including FISH and DNA array for gene copy number [9, 21] RT-PCR for gene expression [9] and IHC and Western analysis [16, 20, 23] for protein expression. This lack of consistency makes direct comparisons between studies difficult. Reagents obtained from varying sources and home-brewed probes and antibodies are also described in these existing studies. If DXL4 has the potential to be used a biomarker in IBC and in other highly metastatic tumors, standardized methods would improve the ease of analysis.

Importantly, high DLX4 expression in IBC cases from an urban African-American population compared to normal breast tissue samples supports previous findings that DLX4 expression is higher in AA women than in Caucasian women [34]. Using this modification of the Allred scoring system [35] developed for scoring hormone receptors with high sensitivity and specificity, tumors from AA women can be looked at on a larger scale potentially identifying DLX4 as a valid biomarker. Though a difficult task due to the lack of specimens from non-Caucasian women, there is great potential for clinical applications, particularly if race and age are addressed as our findings preliminarily implicate both.

To our knowledge, our findings suggesting that higher DLX4 expression is associated with HER2-negative IBC is novel. This can be important for future studies, as HER2-positive status has been shown to be associated with improved outcomes in metastatic IBC [36]. Further mechanistic studies may reveal a direct, or indirect, association between DLX4 and HER2 in IBC.

Our findings from a targeted AA population, as well as HER2 and ER receptor status also supports the idea that determining the etiology of poor prognosis in IBC may be more complex or separate from the traditional markers that attribute risk. In IBC, molecular characteristics that combine to cause increased proliferation and angiogenesis may be causative factors rather than the traditional ideas of high incidence of ER- and HER2 status which serves to oversimplify the IBC profile.

## MATERIALS AND METHODS

### Specimens

Anonymized formalin-fixed paraffin-embedded (FFPE) tissue blocks from well-characterized African-American inflammatory breast cancer (IBC) cases (*n* = 29) were obtained from the Department of Pathology, Howard University Hospital in Washington, DC. Ethical approval was received from Howard University's Institutional Review Board for this study, IRB-15-CMED-53. The cases for this study were collected from 2006–2009 and were selected for further study based upon clinical diagnosis of IBC and availability of FFPE tissue blocks. Slides were graded according to the Nottingham grading system by a Board-Certified Pathologist (TN) [37]. Five μm thick sections were cut from study blocks, and areas of well-preserved IBC in a Hematoxylin and Eosin (H&E) orienting slide from each block was marked for study and scoring after IHC. Normal breast tissue control slides of five μm were prepared from FFPE human breast tissue blocks (*n* = 30) from reduction mammoplasties from subjects with mixed ethnicity. There were no pathologic breast abnormalities. A Board-Certified Pathologist also examined these cases confirming that all were normal breast tissue. These slides were provided by the Georgetown University Medical Center Histology and Tissue Shared Resource (HTSR) in Washington, DC.

### Immunohistochemistry

IHC was performed using the indirect method, as previously described [38]. The primary polyclonal antibody NB100-481 against the human BP1 (DLX4) variant 1 peptide was produced in rabbit from Novus Biologicals USA (Littleton, CO, USA) and previously used in BP-1 research by Fu *et al*. 2010 [32] and Stevenson *et al.* [39]. Hereafter the BP1 antibody will be referred to as DLX4. A horseradish peroxidase (HRP)-conjugated polyclonal goat anti-rabbit immunoglobulins secondary antibody from DAKO North America (Carpinteria, CA, USA) was used for detection. DLX4 antibody was applied at a 1:50 dilution with positive and negative controls included for each trial. Slides of invasive breast ductal carcinoma tissue and normal human tonsil tissue provided by HTSR of Georgetown University Medical Center were used as positive controls and negative controls for adjustment of antibody concentration. Specificity was further assured by antibody substitution with PBS. FFPE tissues were baked overnight at 60° C on a slide warmer and deparaffinized in two Xylene washes for 10 minutes each at room temperature. Ten minutes of incubation in 100% ethanol at room temperature was used a final fixative. Heat induced epitope retrieval (HIER) was performed in 10 mM sodium citrate, pH 6.0, at 100° C for 20 minutes. Exposure to 3% hydrogen peroxide (H_2_O_2_) for 10 minutes at room temperature was used to prevent endogenous peroxidase activity. This was followed by washing in Tris-buffered saline plus 0.05% Tween 20 (TBST), pH 9.0. Ten% normal goat serum in TBST was applied to the sections for 10 minutes at room temperature to eliminate interactions with the goat-derived secondary antibodies. The sections were incubated with the rabbit polyclonal antibody to human DLX4 at a 1:70 dilution for an hour at room temperature. The sections were washed in TBST and treated with the horseradish peroxidase (HRP)-conjugated polyclonal goat anti-rabbit immunoglobulins secondary antibody for 30 minutes at room temperature, followed by washing three times in TBST at room temperature. The tissue-bound HRP-conjugated antibody was stained by 3, 3′-diaminobenzidine (DAB) for 5 minutes at room temperature. The sections were washed in deionized H_2_O and counterstained with hematoxylin for better visualization of the tissue morphology. The sections were then incubated in 1% ammonium hydroxide for 1 minute at room temperature.

### Scoring

IHC results were assigned an intensity and percentage score based upon both intensity of positive staining and number of stained cells, respectively. Percentage scores were assigned as 0, 1 (0%–25%), 2 (26%–50%), 3 (51%–75%) or 4 (76%–100%) and intensity scores were assigned 0, 1+, 2+ or 3+. The status of DLX4 expression among cells in the IBC and control tissues was independently scored according to the Allred method [35] by two Clinical pathologists (T.J.N. and M.S.O), and the average of the score values obtained was used in the statistical analysis.

### Hormone-receptors and Her2/Neu status

The data on estrogen receptor (ER), progesterone receptor (PR) and Human epidermal growth factor receptor type 2 (Her2/Neu) status of the IBC cases were obtained from medical and laboratory records provided by Howard University Hospital. Table 1 shows the characteristics of the patient group. The IBC cases were considered TNBC if the ER marker, the PR marker and HER2 status were negative, and as non-triple negative breast cancer if any marker was positive [40]. Both hormone receptor status and HER2 receptor status were available for all the IBC cases in this study.

### Statistical analysis

For the analysis of the protein expression (IHC), a percentage score of 3 or 4 was considered high, and the score of 0, 1 or 2 low. An intensity score of 2+ or 3+ was categorized as high, and the score of 0 or 1+ low. Chi-square or Fisher's exact tests were used to assess the association between grade expression and DLX4 expression (percentage and intensity), breast cancer clinicopathology findings (ER, PR, HER2, TNBC) and protein expression. Mann–Whitney *U* test were used to compare age between high and low groups of the DLX4 protein expression. All statistical analyses were conducted using IBM SPSS statistics 24 software (IBM Corp., Armonk, NY, USA). *P*-values of 0.05 or less were considered significant.

## Abbreviations

DLX4: Distal-less homeobox 4
IBC: Inflammatory breast cancer
ER: Estrogen receptor
PR: Progesterone receptor
HER2: Human epidermal growth factor receptor type 2
IHC: immunohistochemical staining
TNBC: Triple negative breast cancer.

## References

[1] Anderson WF, Schairer C, Chen BE, Hance KW, Levine PH (2005). Epidemiology of Inflammatory Breast Cancer (IBC). Breast Disease.

[2] Amin MB, Greene FL, Edge SB, Compton CC, Gershenwald JE, Brookland RK, Meyer L, Gress DM, Byrd DR, Winchester DP (2017). The Eighth Edition AJCC Cancer Staging Manual: Continuing to build a bridge from a population-based to a more “personalized” approach to cancer staging. CA: a Cancer Journal for Clinicians.

[3] Fitzgibbons PL, Bose S, Chen YY, Connolly JL, de Baca ME, Edgerton M, Hayes DF, Hill KA, Kleer C, Lester SC, O'Malley FP, Page DL (2018). Protocol for the examination of specimens from patients with invasive carcinoma of the breast: InvasiveBreast 4.1.0.0 Protocol, Posting Date: January 2018 Includes pTNM requirements from the 8th Edition, AJCC Staging Manual For accreditation purposes, this protocol should be used for the following procedures. http://www.cap.org/cancerprotocols.

[4] Lopez MJ, Porter KA (1996). Inflammatory breast cancer. Surgical Clinics of North America.

[5] Low JA, Berman AW, Steinberg SM, Danforth DN, Lippman ME, Swain SM (2004). Long-term follow-up for locally advanced and inflammatory breast cancer patients treated with multimodality therapy. Journal of Clinical Oncology.

[6] Levine PH, Steinhorn SC, Ries LG, Aron JL (1985). Inflammatory breast cancer: The experience of the Surveillance, Epidemiology and End Results (SEER) Program. J Natl Cancer Inst.

[7] American Cancer Society Overview of inflammatory breast cancer. https://www.cancer.org/cancer/breast-cancer/understanding-a-breast-cancer-diagnosis/types-of-breast-cancer/inflammatory-breast-cancer.html.

[8] Hance KW, Anderson WF, Devesa SS, Young HA, Levine PH (2005). Trends in inflammatory breast carcinoma incidence and survival: the surveillance, epidemiology, and end results program at the National Cancer Institute. Journal of the National Cancer Institute.

[9] Parton M, Dowsett M, Ashley S, Hills M, Lowe F, Smith IE (2004). High incidence of HER-2 positivity in inflammatory breast cancer. The Breast.

[10] Sawaki M, Ito Y, Akiyama F, Tokudome N, Horii R, Mizunuma N, Takahashi S, Horikoshi N, Imai T, Nakao A, Kasumi F (2006). High prevalence of HER-2/neu and p53 overexpression in inflammatory breast cancer. Breast Cancer.

[11] Dawood S, Broglio K, Gong Y, Yang WT, Cristofanilli M, Kau SW, Meric-Bernstam F, Buchholz TA, Hortobagyi GN, Gonzalez-Angulo AM (2008). Prognostic significance of HER-2 status in women with inflammatory breast cancer. Cancer.

[12] Zell JA, Tsang WY, Taylor TH, Mehta RS, Anton-Culver H (2009). Prognostic impact of human epidermal growth factor-like receptor 2 and hormone receptor status in inflammatory breast cancer (IBC): analysis of 2,014 IBC patient cases from the California Cancer Registry. Breast Cancer Research.

[13] Zhang L, Yang M, Gan L, He T, Xiao X, Stewart MD, Liu X, Yang L, Zhang T, Zhao Y, Fu J (2012). DLX4 upregulates TWIST and enhances tumor migration, invasion and metastasis. International Journal of Biological Sciences.

[14] Ha KY, Glass SB, Laurie L (2013). Inflammatory breast carcinoma.

[15] Colpaert CG, Vermeulen PB, Benoy I, Soubry A, Van Roy F, Van Beest P, Goovaerts G, Dirix LY, Van Dam P, Fox SB, Harris AL (2003). Inflammatory breast cancer shows angiogenesis with high endothelial proliferation rate and strong E-cadherin expression. British Journal of Cancer.

[16] Van der Auwera I, Van Laere SJ, Van den Eynden GG, Benoy I, van Dam P, Colpaert CG, Fox SB, Turley H, Harris AL, Van Marck EA, Vermeulen PB (2004). Increased angiogenesis and lymphangiogenesis in inflammatory versus noninflammatory breast cancer by real-time reverse transcriptase-PCR gene expression quantification. Clinical cancer research.

[17] Samuel S, Naora H (2005). Homeobox gene expression in cancer: insights from developmental regulation and deregulation. European Journal of Cancer.

[18] Lawrence HJ, Largman C (1992). Homeobox genes in normal hematopoiesis and leukemia. Blood.

[19] Kawai H, Tomii K, Toyooka S, Yano M, Murakami M, Tsukuda K, Shimizu N (2005). Promoter methylation down-regulates CDX2 expression in colorectal carcinomas. Oncology Reports.

[20] Raman V, Martensen SA, Reisman D, Evron E, Odenwald WF, Jaffee E, Marks J, Sukumar S (2000). Compromised HOXA5 function can limit p53 expression in human breast tumours. Nature.

[21] Wimmer K, Zhu XX, Rouillard JM, Ambros PF, Lamb BJ, Kuick R, Eckart M, Weinhäusl A, Fonatsch C, Hanash SM (2002). Combined restriction landmark genomic scanning and virtual genome scans identify a novel human homeobox gene, ALX3, that is hyper-methylated in neuroblastoma. Genes, Chromosomes and Cancer.

[22] Naora H, Montz FJ, Chai CY, Roden RB (2001). Aberrant expression of homeobox gene HOXA7 is associated with müllerian-like differentiation of epithelial ovarian tumors and the generation of a specific autologous antibody response.

[23] Quinn LM, Johnson BV, Nicholl J, Sutherland GR, Kalionis B (1997). Isolation and identification of homeobox genes from the human placenta including a novel member of the Distal-less family, DLX4. Gene.

[24] Wu D, Mandal S, Choi A, Anderson A, Prochazkova M, Perry H, Gil-Da-Silva-Lopes VL, Lao R, Wan E, Tang PL, Kwok PY (2015). DLX4 is associated with orofacial clefting and abnormal jaw development. Human Molecular Genetics.

[25] Fu S, Stevenson H, Strovel JW, Haga SB, Stamberg J, Do K, Berg PE (2001). Distinct functions of two isoforms of a homeobox gene, BP1 and DLX7, in the regulation of the β-globin gene. Gene.

[26] Chase MB, Fu S, Haga SB, Davenport G, Stevenson H, Do K, Morgan D, Mah AL, Berg PE (2002). BP1, a homeodomain-containing isoform of DLX4, represses the β-globin gene. Molecular and Cellular Biology.

[27] Schwartz AM, Man YG, Rezaei MK, Simmens SJ, Berg PE (2009). BP1, a homeoprotein, is significantly expressed in prostate adenocarcinoma and is concordant with prostatic intraepithelial neoplasia. Modern Pathology.

[28] Cavalli LR, Man YG, Schwartz AM, Rone JD, Zhang Y, Urban CA, Lima RS, Haddad BR, Berg PE (2008). Amplification of the BP1 homeobox gene in breast cancer. Cancer Genetics and Cytogenetics.

[29] Fu SW, Schwartz A, Stevenson H, Pinzone JJ, Davenport GJ, Orenstein JM, Gutierrez P, Simmens SJ, Abraham J, Poola I, Stephan DA (2003). Correlation of expression of BP1, a homeobox gene, with estrogen receptor status in breast cancer. Breast Cancer Research.

[30] Man YG, Schwartz A, Levine PH, Teal C, Berg PE (2009). BP1, a putative signature marker for inflammatory breast cancer and tumor aggressiveness. Cancer Biomarkers.

[31] Torresan C, Oliveira MM, Pereira SR, Ribeiro EM, Marian C, Gusev Y, Lima RS, Urban CA, Berg PE, Haddad BR, Cavalli IJ (2014). Increased copy number of the DLX4 homeobox gene in breast axillary lymph node metastasis. Cancer Genetics.

[32] Fu Y, Lian Y, Kim KS, Zhang L, Hindle AK, Brody F, Siegel RS, McCaffrey TA, Fu SW (2010). BP1 homeoprotein enhances metastatic potential in ER-negative breast cancer. J Cancer.

[33] Langa BC, Oliveira MM, Pereira SR, Lupicki K, Marian C, Govender D, Panieri E, Hiss D, Cavalli JI, Abdul-Rasool S, Cavalli LR (2015). Copy Number Analysis of the DLX4 and ERBB2 Genes in South African Breast Cancer Patients. Cytogenetic and Genome Research.

[34] Man YG, Fu SW, Schwartz A, Pinzone JJ, Simmens SJ, Berg PE (2005). Expression of BP1, a novel homeobox gene, correlates with breast cancer progression and invasion. Breast Cancer Research and Teatment.

[35] Allred DC, Bustamante MA, Daniel CO (1990). Immunocytochemical analysis of estrogen receptors in human breast carcinomas. Evaluation of 130 cases and review of the literature regarding concordance with biochemical assay and clinical relevance. Arch Surg.

[36] Weiss A, Menen RS, Lin HY, Shen Y, Rosso KJ, Shaitelman S, Woodward W, Valero V, Ueno NT, Bedrosian I, Babiera G (2018). Factors associated with improved outcomes for metastatic inflammatory breast cancer patients. Breast Cancer Res Treat.

[37] Bloom HJ, Richardson WW (1957). Histological grading and prognosis in breast cancer: a study of 1409 cases of which 359 have been followed for 15 years. British Journal of Cancer.

[38] Taylor CR, Burns J (1974). The demonstration of plasma cells and other immunoglobulin-containing cells in formalin-fixed, paraffin-embedded tissues using peroxidase-labelled antibody. Journal of Clinical Pathology.

[39] Stevenson HS, Fu SW, Pinzone JJ, Rheey J, Simmens SJ, Berg PE (2007). BP1 transcriptionally activates bcl-2and inhibits TNFα-induced cell death in MCF7 breast cancer cells. Breast Cancer Research.

[40] Foulkes WD, Smith IE, Reis-Filho JS (2010). Triple-negative breast cancer. New England Journal of Medicine.

